# *BRAF* mutation is a powerful prognostic factor in advanced and recurrent colorectal cancer

**DOI:** 10.1038/bjc.2011.19

**Published:** 2011-02-01

**Authors:** T Yokota, T Ura, N Shibata, D Takahari, K Shitara, M Nomura, C Kondo, A Mizota, S Utsunomiya, K Muro, Y Yatabe

**Affiliations:** 1 Department of Clinical Oncology, Aichi Cancer Center Hospital, Kanokoden, Chikusa-ku, Nagoya 464-8681, Japan; 2 Department of Pathology and Molecular Diagnostics, Aichi Cancer Center Hospital, Kanokoden, Chikusa-ku, Nagoya 464-8681, Japan; 3 Department of Gastroenterology, Nagoya Kyoritsu Hospital, Nakagawa-ku, Nagoya 454-0933, Japan

**Keywords:** *BRAF*, *KRAS*, prognostic marker, colorectal cancer, chemotherapy

## Abstract

**Background:**

Activating mutation of *KRAS* and *BRAF* are focused on as potential prognostic and predictive biomarkers in patients with colorectal cancer (CRC) treated with anti-EGFR therapies. This study investigated the clinicopathological features and prognostic impact of *KRAS/BRAF* mutation in advanced and recurrent CRC patients.

**Method:**

Patients with advanced and recurrent CRC treated with systemic chemotherapy (*n*=229) were analysed for *KRAS/BRAF* genotypes by cycleave PCR. Prognostic factors associated with survival were identified by univariate and multivariate analyses using the Cox proportional hazards model.

**Results:**

*KRAS* and *BRAF* mutations were present in 34.5% and 6.5% of patients, respectively. *BRAF* mutated tumours were more likely to develop on the right of the colon, and to be of the poorly differentiated adenocarcinoma or mucinous carcinoma, and peritoneal metastasis. The median overall survival (OS) for *BRAF* mutation-positive and *KRAS* 13 mutation-positive patients was 11.0 and 27.7 months, respectively, which was significantly worse than that for patients with wild-type (wt) *KRAS* and *BRAF* (40.6 months) (*BRAF;* HR=4.25, *P*<0.001, *KRAS13;* HR=2.03, *P*=0.024). After adjustment for significant features by multivariate Cox regression analysis, *BRAF* mutation was associated with poor OS (HR=4.23, *P*=0.019).

**Conclusion:**

Presence of mutated *BRAF* is one of the most powerful prognostic factors for advanced and recurrent CRC. The *KRAS13* mutation showed a trend towards poor OS in patients with advanced and recurrent CRC.

Although the epidermal growth factor receptor (EGFR) has important roles in cell differentiation and proliferation in normal cells, activation of EGFR signalling is frequently observed in colorectal cancer (CRC) cells, resulting in cell proliferation, migration and metastasis, evasion of apoptosis, or angiogenesis (Fang and Richardson, 2005). Indeed, ∼35% of CRC tissues carry a mutation in codons 12 or 13 of *KRAS* that leads to the constitutive activation of downstream pathways, including the Ras/Raf/MAP/MEK/ERK and/or PTEN/PI3K/Akt pathways (Kinzler and Vogelstein, 1999; Wan *et al*, 2004; Benvenuti *et al*, 2007; Di Nicolantonio *et al*, 2008; Souglakos *et al*, 2009). *BRAF* is a downstream molecule of *KRAS*. Although more than 40 somatic mutations in the *BRAF* kinase domain have been described, the most common mutation across various cancers is the classic GTG → GAG substitution at the position 1799 of exon 15, which results in the V600E amino acid change, and the subsequent constitutive activation of the EGFR signalling pathway. Recent studies from Western countries have suggested that *BRAF* mutations occur in 10–20% of patients with sporadic disease (Jass, 2007; Benvenuti *et al*, 2007; Di Nicolantonio *et al*, 2008; Souglakos *et al*, 2009; Fariña-Sarasqueta *et al*, 2010), whereas other reports have revealed that tumours harbouring *BRAF* mutations have different clinical and histopathological features compared with tumours that harbour *KRAS* mutations (Kim *et al*, 2006; Deng *et al*, 2008; Zlobec *et al*, 2010). However, the frequency and clinicopathological features of *KRAS/BRAF* mutation in Japanese CRC patients remain unknown.

Information on *KRAS/BRAF* genotype is extremely useful in systemic chemotherapy for advanced and recurrent CRC patients, not just for predicting the therapeutic efficiency of anti-EGFR therapy, but also for identifying patients with poor prognoses. Therefore, both *KRAS* and *BRAF* are currently being focused on as potential prognostic and predictive biomarkers in patients with metastatic disease treated with anti-EGFR therapies, such as panitumumab and cetuximab (Karapetis *et al*, 2008; Bokemeyer *et al*, 2009; Tol *et al*, 2009; Van Cutsem *et al*, 2009). A number of retrospective analyses have revealed that patients with *KRAS* mutations do not benefit from cetuximab treatment, suggesting that *KRAS* genotype is a useful predictive marker for cetuximab therapy in CRC (Karapetis *et al*, 2008; Bokemeyer *et al*, 2009; Van Cutsem *et al*, 2009). It has also been reported that wild-type (wt) *BRAF* is required for a successful response to panitumumab or cetuximab therapies in metastatic CRC (Di Nicolantonio *et al*, 2008; Laurent-Puig *et al*, 2009; Souglakos *et al*, 2009; De Roock *et al*, 2010). In contrast, the prognostic relevance of *KRAS* genotype in CRC has been controversial despite a number of multi-institutional investigations dating from the 1990s (Andreyev *et al*, 1998; French *et al*, 2008; Kakar *et al*, 2008; Ogino *et al*, 2009; Roth *et al*, 2010). Although few studies have investigated the impact of *KRAS12* and *KRAS13* mutations on CRC prognosis, a series of recent studies have highlighted the potential adverse prognostic impact of *BRAF* mutations, using both patients with stage II and III disease and patients across all disease stages (Ogino *et al*, 2009; Fariña-Sarasqueta *et al*, 2010). Although Tol *et al* (2009) analysed *BRAF* genotypes in 520 metastatic CRC patients, all the patients were treated with chemotherapy plus bevacizumab with or without cetuximab. Furthermore, *BRAF* genotypes were analysed in a large subgroup of 845 metastatic CRC treated with FOLFIRI and FOLFOX chemotherapy with or without cetuximab as the first-line treatment in the CRYSTAL and OPUS studies, respectively (Bokemeyer *et al*, 2010). Thus, although the prognostic value of *BRAF* has been analysed in CRC patients treated with specific chemotherapy regimens, it remains unclear what impact the *KRAS12, KRAS13,* and *BRAF* mutations have on clinical outcomes of all patients with advanced or recurrent CRC treated with systemic treatments.

We have previously introduced the cycleave PCR technique as applicable to the routine screening of *KRAS/BRAF* mutations in CRC from pathological specimens, such as surgical and biopsy specimens (Yokota *et al*, 2010). Cycleave PCR utilises chimeric DNA-RNA-DNA probes labelled with a fluorescent dye and quencher, and the accuracy of cycleave PCR in detecting *KRAS/BRAF* mutations has been confirmed by assessment of the concordance between cycleave PCR and reverse transcriptase PCR-coupled direct sequencing (Yatabe *et al*, 2006; Yokota *et al*, 2010).

The aim of this study was to evaluate the *KRAS/BRAF* genotypes of advanced and recurrent CRC patients and to assess the effects of these genotypes on clinical outcome. To this end, we analysed the frequencies of the *KRAS12, KRAS13* and *BRAF* mutations, and correlated these results with the clinicopathological features of 229 Japanese CRC patients.

## Patients and methods

### Patients and tissues

Analysis of the genes encoding *KRAS* and *BRAF* was performed on surgically resected or biopsied specimens from CRC patients at our institution from 2002 to 2010. Hematoxylin and eosin (H and E)-stained slides were retrospectively collected and histologic subtypes were reviewed by an experienced gastrointestinal pathologist. Clinicopathological and survival analyses were subsequently performed on all patients with advanced and recurrent CRC who underwent systemic chemotherapy. Clinical data, including patient age at diagnosis, tumour location, and metastatic sites, were retrieved from patient records. Right-sided cancers included tumours from the caecum to transverse colon, left-sided included tumours from the splenic flexure to the rectosigmoid junction. Specimens used for *KRAS/BRAF* genotyping were either frozen or paraffin embedded tissues. For the *KRAS/BRAF* genotyping, appropriate approvals were obtained from the institutional review committee and written informed consent was obtained from all patients.

### DNA extraction

DNA was extracted from surgical or biopsy specimens. Briefly, tumour cell-rich areas in H and E-stained sections were marked under a microscope, and tissues scratched from the same areas were sequentially deparaffinised and unstained. Recovered tissues were incubated in 1X PCR buffer containing 100 *μ*g ml^−1^ proteinase K for 1 h at 54 °C. After heat inactivation at 95 °C for 3 min, samples were used directly as template DNA for PCR assay.

### *KRAS/BRAF* genotyping by cycleave PCR

To detect point mutations at *KRAS* codons 12, 13 and 61, we used the cycleave PCR technique (Yatabe *et al*, 2006; Sakamoto *et al*, 2007; Yokota *et al*, 2010). Each chimeric DNA-RNA-DNA probe was labelled with a fluorescent dye and quencher at each end that targeted the G12D, G12V, G12R, G12C, G12S, or G12A mutations in codon 12, the G13D or G13C mutations in codon 13, or the G61H, G61L, G61E, or G61K mutations in codon 61 of *KRAS*. We also designed probes that targeted the V600E mutation in *BRAF*. The PCR reactions were performed using a cycleave PCR core kit (TAKARA, Co. Ltd, Ohtsu, Japan). Fluorescent signals were quantified using the Smart Cycler system (SC-100; Cepheid, Sunnyvale, CA, USA).

### Statistical analysis

The *χ*^2^, Fischer's exact tests and Student's *t*-tests were used to analyse the relationship between variables using SYSTAT software (SYSTAT Software Inc., Richmond, CA, USA). The *KRAS* wt/*BRAF* wt (wild/wild), *KRAS12* mutant (G12X), *KRAS13* mutant (G13X), and *BRAF* mutant (V600E) groups were analysed separately. Overall survival (OS) was calculated from the starting date of the first-line chemotherapy until death from any cause, or censored at last follow-up visit. Survival data were analysed using the Kaplan–Meier product-limit method. Comparison of survival curves was carried out using the log-rank test. We first performed a univariate comparison of survival functions for factors that could potentially affect the survival time using the log-rank test, and then a multivariate analysis using the Cox proportional hazards model. *P*-values <0.05 were considered statistically significant, and all *P*-values represent two-sided significance tests.

## Results

### Frequency of *KRAS* and *BRAF* gene mutations in CRC patients

According to our previous investigation on the spectrum of *KRAS* genotypes in our database of CRC cases, the most frequent mutations at *KRAS* codon 12 were the G12D, G12V, G12R, G12C, G12S and G12A mutations, which accounted for more than 95% of the codon 12 mutations. Similarly, the G13D and G13C mutations at codon 13, and the G61H, G61L, G61E, and G61K mutations at codon 61 were also found to be the most common at each site (Yokota *et al*, 2010). All the *KRAS* mutations we located have been previously described as oncogenically active and were present in the COSMIC (catalogue of somatic mutations in cancer) database (Sanger Institute, Cambridge, UK). Therefore, a series of specific probes targeting the common mutations in *KRAS* codons 12, 13 and 61 were designed for subsequent analysis of *KRAS* mutation frequency in our population of CRC patients. Because the most common mutation in *BRAF* is a valine to glutamate transition at position 600 of the protein (V600E), we designed probes targeting the V600E mutation in *BRAF*.

We initially analysed the *KRAS* genotypes of 349 CRC patients at our institution for which pathological specimens were available by cycleave PCR. The *KRAS* mutations were present in 35.7% (*n*=126) of patients tested, including 24.4% (*n*=86) that exhibited codon 12 mutations and 11.3% (*n*=40) that exhibited codon 13 mutations. However, only 4.7% (*n*=15) of the patients tested were positive for the *BRAF* V600E mutation (*n*=319). None of the *KRAS*-mutated samples carried a concomitant *BRAF* mutation. Approximately 2–3% of the surgical specimens could not be evaluated by cycleave PCR, probably due to over-fixation by formalin, as we reported previously (Yokota *et al*, 2010).

For the subsequent clinicopathological and survival analysis, we picked out 229 patients with advanced and recurrent CRC for which we could access complete clinicopathological information. The *KRAS* mutations were present in 34.5% (*n*=79) of advanced and recurrent CRC patients, including 23.1% (*n*=53) with codon 12 mutations and 11.4% (*n*=26) with codon 13 mutations. The *BRAF* mutation was found in 6.6% (*n*=15) of this population (Table 1).

### Association of *BRAF/KRAS* mutations with clinicopathological features

We then correlated the *KRAS* and *BRAF* genotypes with clinicopathological features of CRC, including primary tumour location, histological findings, and sites of metastases. We categorised the population into four subtypes; those with wt *KRAS* and *BRAF* (wild/wild), *KRAS12* mutations (G12X), *KRAS13* mutations (G13X), and *BRAF* mutations (V600E).

For disease status, recurrent disease was more frequent in the *KRAS12* and *KRAS13* mutant groups than in the wild/wild group. There was no association between *KRAS/BRAF* genotype and age, gender or PS. Primary tumours were located at the rectum in almost half of the wild/wild and G12X populations. However, right-side tumour location was more frequent (60%) in patients with *BRAF* mutation in all subtypes (*P*=0.0391) (Table 2). Furthermore, 46.2% (12 out of 26) of the primary tumours with *KRAS13* mutations were located on the right side whereas the frequencies of right-side location were 20.7% (28 out of 135) and 26.4% (14 out of 53), for the wild/wild and G12X groups, respectively (Table 2). The *BRAF* and *KRAS13* mutations were present in 14.3% (9 out of 63) and 19.0% (17 out of 63) of right-sided CRC, respectively. These results suggested that the *BRAF* and *KRAS* codon 13 mutations were associated with a right-sided tumour location.

Analysis with respect to histology showed that the frequencies of poorly differentiated adenocarcinoma (por), mucinous carcinoma (muc) and signet-ring cell carcinoma (sig) were <10.9% in patients with wt *BRAF*, which supported previous reports that such histologies are rare in CRC (Ogino *et al*, 2006; Catalano *et al*, 2009). However, 60.0% (9 out of 15) of CRC cases with *BRAF* mutation were of the por or muc subtypes, although no signet-ring cell carcinomas were observed. The *BRAF* mutations were present in 36.0% (9 out of 25) of patients with por/muc histology. Furthermore, 60.0% (9 out of 15) of CRCs with *BRAF* mutation metastasised to the peritoneum, compared with ∼15% of CRCs with other subtypes (*P*=0.0062) (Table 2). However, Fisher's exact test indicated no statistically significant correlation between tumour histology and peritoneal metastasis in *BRAF* mutant patients. No other significant differences or trends in metastatic patterns with respect to *KRAS/BRAF* genotypes were observed.

Details of the first line chemotherapy regimens used are shown in Table 2. In all, 66.4% of patients were treated with oxaliplatin-based regimens, 14.4% with irinotecan-based regimens, and 19.2% with fluoropyrimidine-based chemotherapy without oxaliplatin or irinotecan. There were no significant differences in treatment regimens between *KRAS/BRAF* genotypes. A total of 86 (63.7%) patients with wild/wild tumours and five (33.3%) patients with *BRAF* mutation-positive tumours received anti-EGFR therapy, whereas few patients with *KRAS12* or *KRAS13* mutations received anti-EGFR therapy (1.9% and 3.8%, respectively).

### Survival

**Figure 1:**
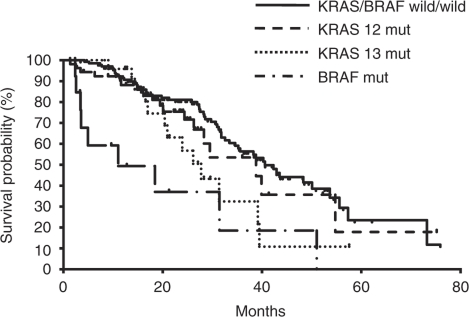
Kaplan–Meier plot showing overall survival in metastatic and recurrent colon cancer patients according to *KRAS* and *BRAF* V600E mutational status (*n*=229). mut, mutated.

The median OS for *BRAF* mutation-positive patients was 11.0 months, which was significantly worse than for patients with wt *KRAS* and *BRAF* (40.6 months) (HR=4.25, 95% CI 2.08–8.67, *P*<0.001; Figure 1). The median OS for all *KRAS* mutation-positive patients, including those with *KRAS12* or *KRAS13* mutations, was not statistically different to that of wt *KRAS* and *BRAF* patients (HR=1.51, 95% CI 0.97–2.36, *P*=0.071). However, if OS for *KRAS13* mutation-positive patients was analysed separately from *KRAS12* mutation-positive patients, then the median OS for *KRAS13* mutation-positive patients was significantly worse than that for wt *KRAS* and *BRAF* patients (27.7 months *vs* 40.6 months, HR=2.03, 95% CI 1.10–3.74, *P*=0.024; Figure 1). In contrast, the median OS for *KRAS12* mutation-positive patients was 38.8 months, similar to that for wt *KRAS* and *BRAF* patients (HR=1.28, 95% CI 0.74–2.19, *P*=0.376; Figure 1). Univariate analysis showed that two other variables were also significantly associated with poor survival, PS ECOG⩾2 and gender (Table 3). *KRAS13* mutation was not statistically associated with poor survival by univariate analysis. This was because we compared OS for *KRAS13* mutation-positive patients with that for wt *KRAS13* patients, which included *KRAS12* and *BRAF* mutation-positive patients as well as wt *KRAS* and *BRAF* patients. The por/sig/muc histology and lung metastasis showed a trend towards poor OS (*P*=0.066 and *P*=0.061, respectively).

To correct for significant prognostic factors, a Cox proportional hazards model that included age, gender, PS, *KRAS* status, *BRAF* status, pathological finding, number of metastasis and metastatic sites, was used. As two variables, WBC and ALP, had missing data, they were not included in the multivariate analysis. *BRAF* mutation and PS ECOG⩾2 were confirmed as poor prognostic factors. Specifically, the relative risk of death for patients with *BRAF* mutation was 4.23 (95% CI 1.76–10.2) compared with patients with wt *BRAF* tumours (*P*=0.001) (Table 3). Multivariate analysis also found that por/sig/muc histology, age>65, and liver metastasis were negative independent prognostic factors. However, *KRAS13* mutation was not found to be an independent prognostic factor.

## Discussion

In this study, we examined the incidence of *KRAS* and *BRAF* mutations in advanced and recurrent CRC patients, and clarified the relationship between *KRAS/BRAF* genotypes and clinicopathological features, including survival. Up to now, estimates of *KRAS* gene mutation frequency in metastatic CRCs have been based on selective clinical studies or drug admission trials with variable inclusion criteria. To our knowledge, the present report is the first to provide data on the frequency and type of *KRAS/BRAF* mutations from a large Japanese population of advanced and recurrent CRC patients tested in a routine setting.

Our results showed that *KRAS* mutation was observed in around 35% of CRC cases, which included 25% of patients with mutations at codon 12 and 10% of patients with mutations at codon 13. This observation agreed well with previous studies on selected cohorts that reported frequencies in the range of 30–42% (Table 1). The cycleave PCR technique was simultaneously applied to the detection of *BRAF* mutation, thought to be an adverse prognostic marker as well as a predictive marker for anti-EGFR therapy. Our analysis demonstrated that the *BRAF* V600E mutation was observed in ∼5% of CRC patients, which appeared to be lower than that previously reported from Western countries. None of the CRC patients in our study carried both *KRAS* and *BRAF* mutations, supporting the hypothesis that *KRAS* and *BRAF* mutations occur in a mutually exclusive manner (Rajagopalan *et al*, 2002; Frattini *et al*, 2004; Ahlquist *et al*, 2008). One possible explanation for the comparatively low frequency of *BRAF* mutation might be the different ethnic group. Indeed, several studies have reported that the mutation rates of DNA mismatch repair (MMR) genes, such as *hMSH2* and *hMLH1*, in hereditary non-polyposis colorectal cancer, is variable between countries. Therefore, geographical variation may account for differences in the mutation spectrum of *BRAF*, as observed for MMR genes (Wei *et al*, 2003; Lee *et al*, 2005; Goldberg *et al*, 2008).

We also investigated the clinicopathological characteristics of CRC patients with respect to *KRAS12, KRAS13* and *BRAF* mutations. In accordance with previous reports (Kim *et al*, 2006; Deng *et al*, 2008; Zlobec *et al*, 2010), *BRAF* mutation occurred more frequently in right-sided tumour locations. We also found that 60.0% of the *BRAF* mutation-positive specimens were of the poorly differentiated adenocarcinoma or mucinous carcinoma subtypes. It was recently reported that mucinous histology predicts a poor response to oxaliplatin- and/or irinotecan-based chemotherapies and is correlated with poor OS (Catalano *et al*, 2009). As *BRAF* mutation was more frequent in mucinous groups than non-mucinous carcinoma, as demonstrated by the present study and others (Ogino *et al*, 2006), the poor prognosis associated with mucinous histology may be at least partially explained by *BRAF* gene mutation. These specific clinicopathological features support the hypothesis that the *BRAF* mutation-mediated carcinogenesis in CRC is initiated by altered *BRAF* function as an early step in the serrated pathway (Bennecke *et al*, 2010), leading to activation of RAF-MEK-ERK-MAP signalling.

In contrast to *BRAF* mutation, no significant differences in clinicopathological parameters were observed according to *KRAS* genotype. However, our analysis did suggest that *KRAS13* mutations were also associated with right-sided tumour location. This result raises the possibility that *KRAS13* may have a distinct phenotype from that of other *KRAS* genotypes.

Using a representative cohort of 229 sporadic CRCs, we identified the *BRAF* V600E mutation as an independent prognostic factor for survival in patients with advanced and recurrent CRC. The presence of the *BRAF* mutation is associated with a significantly higher risk of dying of cancer-related causes, independently of other factors such as age, gender, PS, *KRAS* status, pathological finding, number of metastasis and metastatic sites, in agreement with other recent studies (Ogino *et al*, 2009; Tol *et al*, 2009; Bokemeyer *et al*, 2010; Fariña-Sarasqueta *et al*, 2010). For example, analysis of stage II and stage III CRC patients (Fariña-Sarasqueta *et al*, 2010) was consistent with the finding that 44% of our population included recurrent disease. The *BRAF* mutation was correlated with survival in a heterogeneous group of CRC patients that included all disease stages (Ogino *et al*, 2009). Furthermore, a positive correlation between *BRAF* mutation and shorter survival was demonstrated in a homogeneous group of metastatic CRC patients treated with a specific chemotherapy regimen with or without cetuximab (Tol *et al*, 2009; Bokemeyer *et al*, 2010). However, our study focused on the advanced and recurrent group who received systemic chemotherapy, including fluoropyrimidines, in combination with oxaliplatin, irinotecan, bevacizumab and anti-EGFR antibody in several lines. Even though all of the patients in our study received systemic chemotherapy, a positive correlation between *BRAF* mutation and shorter survival was still demonstrated, independent of treatment arm.

The prognostic value of *KRAS* mutations in CRC remains controversial, even though *KRAS* mutations have been associated with a poor response to anti-EGFR antibody therapy in metastatic CRC (Karapetis *et al*, 2008; Bokemeyer *et al*, 2009; Van Cutsem *et al*, 2009). Despite a number of studies investigating a prognostic role for *KRAS* mutations, no definitive conclusions can be drawn (Castagnola and Giaretti, 2005). This may be due to differences between the studies in terms of study size, patient selection, tumour sampling, use of archival versus fresh/frozen material, or laboratory methods and data analyses. More importantly, few studies have differentiated *KRAS* mutations at codon 12 from those at codon 13 with respect to clinicopathological features and survival (Bazan *et al*, 2002). Our analysis revealed that mutation at *KRAS12* had no effect on patient OS. In contrast, our Kaplan–Meier curves clearly demonstrated that OS for patients with *KRAS13* mutations were significantly worse than for those who had wt *KRAS* and *BRAF*. It has been reported that stage III patients with *KRAS* mutations displayed significantly worse disease-free survival, as compared with those with wt *KRAS* (Fariña-Sarasqueta *et al*, 2010). This finding may be partially explained by the impact of *KRAS13* mutations on prognosis. As both univariate and multivariate analysis failed to confirm *KRAS13* mutation as an independent prognostic factor, the prognostic value of mutations at *KRAS13* remains unclear in advanced and recurrent CRC. In non-small-cell lung cancer there are differences in transforming potential and EGFR tyrosine kinase inhibitor sensitivity associated with EGFR somatic mutations L858R and deletion mutant Del (746-750) (Carey *et al*, 2006). Therefore, it remains a possibility that the different *KRAS* mutations at codons 12 and 13 may have different biological consequences that could influence the prognosis for CRC.

With respect to technical issue on *KRAS* and *BRAF* genotyping, we evaluated the prognostic value of the mutations frequently found in *KRAS* and *BRAF* using specific PCR probes. In contrast, direct sequencing is able to detect all possible *KRAS* and *BRAF* mutations including some more rare mutations. In fact, it is reported that *KRAS* codon 146 mutation, which was identified by direct sequencing, was associated with resistance to cetuximab plus irinotecan therapy although this is a minor oncogenic *KRAS* mutation (Loupakis *et al*, 2009). Therefore, direct sequencing may be able to obtain further insights into predictive and prognostic impact of these mutations.

Our study found that the median OS of patients with wt *BRAF* was generally longer than that observed in other reports. It could be argued that the selection of patients with good prognosis could bias the results in this study. Indeed, more than half of our study population was screened for *KRAS/BRAF* genotype to determine the use of anti-EGFR antibody, and 42% of the patients were treated with cetuximab combined therapy mostly as a second- or third-line chemotherapy. Although treatment selection may be a major reason for the longer survival observed in the present study as compared with previous studies involving metastatic CRC patients, univariate analysis revealed no significant differences in survival between patients with and without anti-EGFR therapy (38.8 months *vs* 32.6 months, *P*=0.277) (Table 3). Furthermore, almost all recurrent and advanced CRC patients are routinely screened for *KRAS/BRAF* genotype at the initiation of the first line chemotherapy in our institution since the use of cetuximab was approved for the treatment of CRC patients in Japan.

Another key point of discussion is the potential treatment bias in this retrospective analysis. The focus of the present study is the patient group with advanced and recurrent CRC who received systemic chemotherapy. However, we need to take the difference in the specific treatment regimen among four genotypes into consideration. In particular, 63.7% (86 out of 135) of wt *KRAS* and *BRAF* patients have received anti-EGFR therapy whereas 33.3% (6 out of 15) and 2.5% (2 out of 79) of patients with *BRAF* and *KRAS12/13* mutations have received anti-EGFR therapy, respectively. Therefore, the prognostic advantage of wt *KRAS* and *BRAF* patients over *BRAF* or *KRAS13* mutation might be partially explained by the presence of anti-EGFR therapy. Nevertheless, it is noteworthy that the prognosis of wt *KRAS* and *BRAF* patients was similar to that of the patients with *KRAS12* mutation despite the frequent use of anti-EGFR therapy.

In conclusion, our retrospective analysis demonstrated that *BRAF* mutation was an independent prognostic factor in advanced and recurrent CRC. Although the presence of *KRAS12* mutation had no apparent effect on OS in advanced and recurrent disease, the prognostic value of *KRAS13* mutation remains uncertain. Our results are useful not only for predicting the efficacy of anti-EGFR therapy, but also for identifying patients with shorter OS in response to systemic chemotherapy, regardless of the use of anti-EGFR therapy. The exact effects of *KRAS12* and *KRAS13* mutations on survival require further study. The application of novel strategies targeting *BRAF* kinase is warranted for the treatment of CRC patients with *BRAF* mutation.

## Figures and Tables

**Table 1:** Spectrum of *KRAS/BRAF* mutations in CRC

	**KRAS**			
**BRAF**	**Wild type**	**G12**	**G13**	**61**
**Wild type**	135	53	26	0
**V600E**	15	0	0	0

Abbreviation: CRC=colorectal cancer.

*n*=229.

**Table 2:** Association of *BRAF* and *KRAS* mutational status with clinicopathological features in colorectal cancer

**KRAS/BRAF status**	**Wild/wild**	**KRAS mutant**		**BRAF mutant**			
**Clinicopathological features**	***n*=135**	**G12X *n*=53**	**G13X *n*=26**	**Total (G12X+G13X) *n*=79**	**V600E *n*=15**	*P-value	**Overall *n*=229**
Age at diagnosis (median)	62 (27–83)	62 (40–85)	68 (41–79)	63 (40–85)	62 (30–80)		
							
*Gender*							
Female	47 (34.8%)	27 (50.9%)	13 (50.0%)	40 (50.6%)	8 (53.3%)	0.1082	95
Male	88 (65.2%)	26 (49.1%)	13 (50.0%)	39 (49.4%)	7 (46.7%)		134
							
*ECOG PS*							
0–1	115 (85.2%)	46 (86.8%)	22 (84.6%)	68 (86.1%)	13 (86.7%)	0.7898	196
>2	9 (6.7%)	4 (7.5%)	3 (11.5%)	7 (8.9%)	2 (13.3%)		18
Unknown	11 (8.1%)	3 (5.7%)	1 (3.8%)	4 (5.1%)	0 (0.0%)		15
							
*Tumour location*							
Right sided	28 (20.7%)	14 (26.4%)	12 (46.2%)	26 (32.9%)	9 (60.0%)	0.0391	63
Left sided	41 (30.4%)	13 (24.5%)	3 (11.5%)	16 (20.3%)	3 (20.0%)		60
Rectum	64 (47.4%)	25 (47.2%)	11 (42.3%)	36 (45.6%)	3 (20.0%)		103
Other	2 (1.5%)	1 (1.9%)	0 (0.0%)	1 (1.3%)	0 (0.0%)		3
							
*Disease status*							
Advanced	82 (60.7%)	26 (49.1%)	11 (42.3%)	37 (46.8%)	9 (60.0%)	0.2269	128
Recurrence	53 (39.3%)	27 (50.9 %)	15 (57.7%)	42 (53.2%)	6 (40.0%)		101
							
*Histological subtype*							
Well	28 (20.7%)	8 (15.1%)	7 (26.9%)	15 (19.0%)	1 (6.7%)	<0.0001	44
Mod	91 (67.4%)	37 (69.8%)	18 (69.2%)	55 (69.6%)	5 (33.3%)		151
por/sig/muc	10 (7.4%)	5 (9.4%)	1 (3.8%)	6 (7.6%)	9 (60.0%)		25
Other	1 (0.7%)	0 (0.0%)	0 (0.0%)	0 (0%)	0 (0.0%)		1
Unknown	5 (3.7%)	3 (5.7%)	0 (0.0%)	3 (3.8%)	0 (0.0%)		8
							
*Metastatic sites*							
Liver	90 (66.7%)	31 (58.5%)	15 (57.7 %)	46 (58.2%)	10 (66.7%)	0.6595	146
Peritoneum	30 (22.2%)	11 (20.8%)	4 (15.4%)	15 (20.0%)	9 (60.0%)	0.0062	54
Lung	42 (31.1%)	21 (39.6%)	10 (38.5%)	31 (39.2%)	5 (33.3%)	0.6867	78
CNS	1 (0.7%)	0 (0.0%)	1 (3.8%)	1 (1.3%)	0 (0.0%)	0.3503	2
Bone	9 (6.7%)	3 (5.7%)	2 (7.7%)	5 (6.3%)	2 (13.3%)	0.7736	16
							
*Number of metastatic sites*							
>2	64 (47.4%)	23 (43.4%)	14 (53.8%)	37 (46.8%)	10 (66.7%)	0.4078	111
<1	71 (52.6%)	30 (56.6%)	12 (46.2%)	42 (53.2%)	5 (33.3%)		118
							
*WBC*							
WBC>10000	9 (6.7%)	4 (7.5%)	2 (7.7%)	6 (7.6%)	0 (0.0%)	0.7622	15
WNL	100 (74.1%)	38 (71.7%)	20 (76.9%)	58 (73.4%)	14 (93.3%)		172
Unknown	26 (19.3%)	11 (20.8%)	4 (15.4%)	15 (20.2%)	1 (6.7%)		42
							
*ALP*							
ALP>300	59 (43.7%)	18 (34.0%)	12 (46.2%)	30 (38.0%)	6 (40.0%)	0.6635	95
WNL	49 (36.3%)	24 (45.3%)	10 (38.5%)	34 (43.0%)	8 (53.3%)		91
Unknown	27 (20.0%)	11 (20.8%)	4 (15.4%)	15 (20.0%)	1 (6.7%)		43
							
*First-line regimen*							
IRI-based	24 (17.8%)	6 (11.3%)	2 (7.7%)	8 (10.1%)	1 (6.7%)	0.4062	33
OXA-based	85 (63.0%)	37 (69.8%)	17 (65.4%)	54 (68.4%)	13 (86.7%)		152
Others	26 (19.3%)	10 (18.9%)	7 (26.9%)	17 (21.5%)	1 (6.7%)		44
							
*Anti-EGFR treatment*							
Yes	86 (63.7%)	1 (1.9%)	1 (3.8%)	2 (2.5%)	5 (33.3%)	<0.0001	93
No	44 (32.6%)	52 (98.1%)	25 (96.2%)	77 (97.5%)	10 (66.7%)		131
Unknown	5 (3.7%)	0 (0%)	0 (0%)	0 (0%)	0 (0%)		5

Abbreviations: CNS=central nervous system; ECOG=Eastern Cooperative Oncology Group; EGFR=epidermal growth factor receptor; PS=performance status; well=well-differentiated adenocarcinoma; mod=moderately differentiated adenocarcinoma; por=poorly differentiated adenocarcinoma; muc=mucinous carcinoma; sig=signet-ring cell carcinoma; CNS=central nervous system; IRI=irinotecan; OXA=oxaliplatin, ALP=alkaline phosphatase; WNL=within normal range; WBC=white blood cells.

Patients with both wild-type *KRAS* and wild-type *BRAF* were designated as wild/wild. All patients with *KRAS* mutations (*n*=79) either in codon 12 (G12X) or in codon 13 (G13X) are shown as total (G12X+G13X).

^*^P-values calculated between wild-type *KRAS* and *BRAF* (wild/wild), *KRAS12* mutant (G12X), *KRAS13* mutant (G13X), and *BRAF* mutant (V600E) groups.

**Table 3:** Factors associated with overall survival in univariate and multivariate analyses

	**Univariate analysis**		**Multivariate analysis**	
**Variable**	**Hazard ratio (95% CI)**	***P*-value**	**Hazard ratio (95% CI)**	***P*-value**
Age >65	0.74 (0.48–1.13)	0.157	0.55 (0.34–0.90)	0.018
Female	1.59 (1.06–2.37)	0.025	1.35 (0.85–2.12)	0.201
PS (ECOG)⩾2	6.14 (3.15–12.0)	<0.001	7.66 (3.68–16.0)	<0.001
BRAF mutant	3.78 (1.89–7.54)	<0.001	4.23 (1.76–10.2)	0.001
KRAS 12 mutant	1.03 (0.62–1.74)	0.897	1.57 (0.88–2.81)	0.128
KRAS 13 mutant	1.67 (0.93–3.02)	0.086	1.51 (0.76–2.98)	0.239
Pathology, por/sig/muc	1.74 (0.96–3.14)	0.066	2.38 (1.16–4.90)	0.018
Number of metastasis ⩾2	0.93 (0.63–1.40)	0.738	1.12 (0.61–2.05)	0.714
Liver metastasis	1.36 (0.88–2.11)	0.162	1.72 (1.02–2.90)	0.042
Lung metastasis	0.66 (0.42–1.02)	0.061	0.59 (0.32–1.11)	0.100
Peritoneal metastasis	1.21 (0.76–1.93)	0.417	1.56 (0.85–2.88)	0.154
WBC ⩾10000	1.27 (0.51–3.15)	0.605	—	—
ALP ⩾300	1.21 (0.78–1.88)	0.395	—	—
Anti-EGFR treatment	0.80 (0.53–1.20)	0.277	—	—

Abbreviations: ALP=alkaline phosphatase; PS=performance status; ECOG=Eastern Cooperative Oncology Group; EGFR=epidermal growth factor receptor; por=poorly differentiated adenocarcinoma; muc=mucinous carcinoma; sig=signet-ring cell carcinoma; CI=confidence interval; WBC=white blood cells.
